# Where next for the reproducibility agenda in computational biology?

**DOI:** 10.1186/s12918-016-0288-x

**Published:** 2016-07-15

**Authors:** Joanna Lewis, Charles E. Breeze, Jane Charlesworth, Oliver J. Maclaren, Jonathan Cooper

**Affiliations:** Centre for Maths and Physics in the Life Sciences and Experimental Biology, University College London, Physics Building, Gower Place, London, WC1E 6BT UK; NIHR Health Protection Research Unit in Modelling Methodology, Department of Infectious Disease Epidemiology, Imperial College London, St Mary’s Campus, Norfolk Place, London, W2 1PG UK; UCL Cancer Institute, University College London, 72 Huntley St, London, WC1E 6DD UK; Department of Genetics, University of Cambridge, Downing Street, Cambridge, CB2 3EH UK; Department of Mathematics, University of Auckland, Auckland, 1142 New Zealand; Department of Engineering Science, University of Auckland, Auckland, 1142 New Zealand; Department of Computer Science, University of Oxford, Wolfson Building, Parks Road, Oxford, OX1 3QD UK

**Keywords:** Reproducibility, Replicability, Extensibility, Communication, Policy, Education

## Abstract

**Background:**

The concept of reproducibility is a foundation of the scientific method. With the arrival of fast and powerful computers over the last few decades, there has been an explosion of results based on complex computational analyses and simulations. The reproducibility of these results has been addressed mainly in terms of exact *replicability* or numerical equivalence, ignoring the wider issue of the reproducibility of conclusions through equivalent, extended or alternative methods.

**Results:**

We use case studies from our own research experience to illustrate how concepts of reproducibility might be applied in computational biology. Several fields have developed ‘minimum information’ checklists to support the full reporting of computational simulations, analyses and results, and standardised data formats and model description languages can facilitate the use of multiple systems to address the same research question. We note the importance of defining the key features of a result to be reproduced, and the expected agreement between original and subsequent results. Dynamic, updatable tools for publishing methods and results are becoming increasingly common, but sometimes come at the cost of clear communication. In general, the reproducibility of computational research is improving but would benefit from additional resources and incentives.

**Conclusions:**

We conclude with a series of linked recommendations for improving reproducibility in computational biology through communication, policy, education and research practice. More reproducible research will lead to higher quality conclusions, deeper understanding and more valuable knowledge.

## Background

Reproducibility is a fundamental concept in the philosophy and practice of science. For hundreds of years, care has been taken over the reliability of experimental methods and results. As computation becomes integrated with the experimental and statistical sciences, questions arise about how the classical standards of reproducibility might also apply to computational science [1]. What do we mean by terms such as “reproducible” and “replicable” in this context? Can they be meaningfully applied to complex computational tools, as well as the day-to-day tasks of data analysis? How do we gain confidence in a computational result, showing it is not a fluke or a quirk of a particular setup, and how do we rule out competing explanations? Many of these questions are as yet unanswered, and it has even been suggested that computational science does not currently qualify as a branch of the scientific method because it cannot yet be said to generate reproducible, verifiable knowledge [2].

The most basic form of reproducibility is *replicability*: as Titus Brown neatly expresses it, do “other people get exactly the same results when doing exactly the same thing?” [3] This concept transfers naturally from the experimental sciences, in which even the most basic training emphasises the need for recording details that will allow others (or the original researcher) to repeat experiments at a later date. The discussion of reproducibility in computational research has so far focussed almost exclusively on this aspect.

However, replicability should be only the very minimum standard. The next stage is true *reproducibility* [4]: does “something similar happen in other people’s hands?” [3] This is a more difficult demand to meet, not least because it is less well defined. The question of what we mean by “something similar” is very seldom discussed, let alone reported in research papers. But true reproducibility makes a stronger and more important statement than replicability alone: not only the method, but the phenomenon itself can be reproduced. This is, in fact, closer to what is meant by reproducibility in the experimental sciences, and usually a more relevant point to make. Another aspect of this reproducibility is the ruling out of competing explanations for a result. Experimental scientists might confirm the validity of a conclusion by designing a different experiment which tests the same hypothesis by a different route [5]. In the same way, computational researchers can improve confidence in a result by trying to reach the same conclusion in a different way.

Finally, the underlying aim of reproducing a result is very often to build on the work, rather than just to confirm it. In designing software for reproducible results, then, it also makes sense for researchers to take into account the *extensibility* of their computational method [6].

To gain insights into these three aspects: replicability, reproducibility and extensibility, we consider three case studies drawn from our experience across computational biology: one in software tools for bioinformatics and two in data analysis, simulation and inference studies. By examining the three aspects in each case study, we aim to identify common features of the evolving “reproducibility agenda” that are typically covered neither in purely experimental discussions, nor by studies strictly concerned with software best practice. Although other properties of good software – availability, usability, functionality, etc. – are clearly important, we focus on these three aspects which we propose as a composite definition of reproducibility. Well-designed software can facilitate the three aspects of reproducibility through its good design properties [7, 8]. We conclude by making some recommendations for the community – researchers, peer reviewers, scientific publishers and funders – to allow computational researchers to go beyond replicability to true reproducibility, improving the quality, confidence and value of our work.

## Results

### Software tools for bioinformatics

Bioinformatics is one important area in which complex computational analyses are applied to biological data. We first discuss how our three aspects of reproducibility apply generally in this field, before considering a specific case study.

#### Replicability in bioinformatics

A number of problems can stand in the way of replicating bioinformatics analyses from publications. First, obtaining raw data is often difficult. In theory, public databases should aid replicability and data sharing, but in practice missing metadata often renders this impossible because of the lack of standards for documenting computational analyses [9]. Bioinformatics pipelines for typical sequence-based analysis may include many tools and preprocessing steps which are often unpublished or poorly documented – for example simply stating that data was “analysed using in-house Perl scripts” [10]. Encouragingly, “minimum information” checklists are being developed, and brought together under the umbrella of the Minimum Information for Biological and Biomedical Investigations (MIBBI) project [11], to help researchers ensure they provide all relevant context for their data. The wide variety of data formats used in bioinformatics software can also make replicability hard to achieve, and standards for data exchange and analysis such as the PSI-MI specification for proteomics [12, 13] and the BioPAX language for pathway data [14, 15] have been developed to improve this.

Some bioinformatics tools – for example, the Bowtie aligner [16, 17] – have elements of stochasticity to their method, or may implement statistical tests differently “under the hood” in ways that are not immediately apparent, especially if they are part of a pipeline encoded in a software package. Making clear what tools, options and parameter combinations were used at each step of an analysis aids replication by controlling for these software variables, and minimum information checklists may also be helpful here.

Titus Brown offers comprehensive guidelines to writing a replicable bioinformatics paper [3], including publishing the version-controlled code and data necessary to re-run an analysis and generate figures. The Workflow4Ever project [18] offers another approach to the same aim, and best-practice criteria have also been proposed for computational scientists more broadly [19]. It is worth noting, however, that Brown is an experienced bioinformatician with a self-reported 25 years of coding experience. His suggestions are good aspirational targets, but good software practice requires an initial investment of learning time. A more manageable aspiration, simply copying figures/results and the code used to generate them into a single document, could be an excellent start towards replicable research. As a project progresses and a researcher’s coding practice improves, improved documentation and “added value” can be built up steadily and naturally.

Ensuring replicability can have very real benefits for the progress of science. As an example, a surprising, high-profile result in gene regulation [20, 21] was recently challenged by reanalysis of gene expression data [22]. This would not have been possible without excellent reporting, including complete data and full description of the original analysis.

#### Reproducibility in bioinformatics

Repeating analyses with sufficient data to get an understanding of any variability or bias inherent in an analysis system is important for true reproducibility. For example, sampling biases may influence genetic association studies, so significant associations must be validated in populations that are different from the one originally sampled before they can be deemed to have been reproduced [23]. In these studies it is also worth noting that reproducibility is more useful than replication because replication of results is both difficult and not necessarily an indication that a result is to be believed [24]. More generally, benchmarking different tools on simulated data, where you know the answer you expect to get, would be one way to demonstrate that a tool performs reproducibly alongside existing methods. This is not currently common practice in bioinformatics, although it is well established in other areas including machine learning [25, 26]. Benchmark datasets, like wet lab experiments, should include both positive and negative controls.

For reproducibility comparisons between results to be meaningful, it must be made clear that the same question is being asked in both analyses. The Software Ontology [27] provides a tool for managing this kind of information by describing software’s inputs, outputs and processing tasks. Standards for data exchange and analysis as described above also facilitate the application of methods to different datasets.

#### Extensibility in bioinformatics

A number of bioinformatics tools have been developed collaboratively as one group extended the work of another: for example, the Bioconductor [28] and Galaxy [29–31] projects, and software based on the Bowtie tool [16, 17]. The Beast2 tool for phylogenetic analysis specifically emphasises modularity in its implementation, in order to be extensible by users [32].

Education, networking and support for professional development are important aspects of ensuring the reuse and extension of software. In bioinformatics, as sequencing costs drop, and as sequencing is adopted in clinical settings, more labs are running sequencing experiments. But senior cell biology investigators often lack bioinformatics expertise, meaning that clear communication is crucial to allow wet-lab and computational biologists to communicate and collaborate. This is also essential when we consider that computational biology is often a cyclical process, with new analyses suggesting additional wet-lab experimentation and validation, and further experiments lending themselves to new analyses [33]. Good communication and explanation take precious time and resources, and central investment in training could improve mutual understanding in a time- and cost-efficient way. To this end, the Global Organisation for Bioinformatics Learning, Education & Training (GOBLET) provides a portal for sharing training materials and opportunities [34, 35].

#### Case Study 1: eFORGE, a software tool for bioinformatics

As an example of a bioinformatics tool, we consider eFORGE [36] (manuscript submitted), software written in Perl for extracting functional significance from data produced in Epigenome-Wide Association Studies (EWAS).

##### eFORGE: replicability

The first step in eFORGE replicability is installation on the replicator’s system. It is recommended that software authors test installation on all the main systems their colleagues will use. Practical installation advice is essential to replicable research, and also encourages the dissemination and use of tools. Problems related to locating third-party dependencies can often arise during installation, mainly due to differences in directory structure, and ideally installation should be robust to differences in directory structure, but failing this a clear, possibly visual, explanation of the required structure is important. When complex directory structures are absolutely necessary, tools such as Docker [37–39] and Virtual Machines allow for simpler software installation [40].

Having facilitated the successful installation of a tool, we face the question of assessing replicability: whether the software reproduces the same result, given the same input. For example, replicate runs of eFORGE on the same data give different results because the program includes an element of randomness, so even here we do not expect exact replication of results (except where random number generators have been seeded identically for each run). Benchmarks for replicability should be given, and must take this into account. For example, a script could be provided that runs benchmarks with known seeds – checking exact replication – or repeatedly with random seeds – checking the results have statistical properties expected.

##### eFORGE: reproducibility

The elements of randomness inherent in eFORGE mean that even with identical input, we begin to test true reproducibility rather than exact replicability. At a broader level, eFORGE’s output is currently being investigated given a number of differing but comparable EWAS datasets. A criterion for true reproducibility could then be that the use of different datasets leads to similar conclusions. Software developers might choose to provide centralised records of these investigations by different researchers, giving more validity both to the software and to the underlying science. Centralised records could also provide a means for curating independent software checks, comparisons of results obtained using different software, and conclusions reached through different analyses.

##### eFORGE: extensibility

Extendable software is available, understandable and well annotated. In order to improve understandability, the eFORGE developers provide a webpage in addition to a traditional paper. There they explain both scientific and technical aspects of the software, as well as including eFORGE as a platform-independent web tool [36]. For local installations, the eFORGE code is publicly available in an online repository [41], and the database it requires can be downloaded from the eFORGE webpage [36].

The code annotation consists of a comprehensive “Perl Pod” – the official manual included at the beginning of the code. eFORGE also has many lines of intra-code annotation that complement, but do not replace, the original manual. This intra-code annotation aids understanding of the whole code and each function separately and, importantly, also encourages extension.

These features of the eFORGE software are provided in an attempt to enable the generation of similar tools for other scenarios, reducing the time and cost of applying the analysis to other datasets and promoting the reuse and exchange of the software.

### Reproducibility in data analysis, simulation and inference studies

Another major activity in computational biology is simulating the behaviour of natural systems from mechanistically-based mathematical and computational models. One reason for conducting such simulations is to examine their behaviour under different sets of parameter values, and perhaps to infer parameter values by comparing results to experimental data.

#### Replicability in data analysis, simulation and inference

In simple deterministic studies, replicability is often relatively straightforward to achieve. For example, if a model consists of a system of ordinary differential equations (ODEs) the equations, parameter values and initial conditions should in theory be enough to reproduce the solution. In practice, however, replication is considerably easier and faster if the authors also supply details of the process of simulation (for example, the ODE solvers used, their versions and parameters). The simplest and most complete way to do this is to supply the code used to generate the results in the paper, and hearteningly we find this is increasingly often the case (e.g. [42].) The aim here is not primarily to provide readers with the opportunity to reproduce results exactly, although this might be a useful additional benefit. Rather, it helps ensure that all the information necessary to produce the results is available.

Additional tools for ensuring replicability are publication ‘checklists’ for complete reporting of computational studies, including the Minimum Information Required In Annotation of Models (MIRIAM) [43] and Minimum Information About a Simulation Experiment (MIASE) [44] guidelines. The Computational Modelling in Biology Network (COMBINE) [45] aims to co-ordinate standards and formats for model descriptions, including modelling markup languages such as the Systems Biology Markup Language (SBML) [46] and the Simulation Experiment Description Markup Language (SED-ML) [47]. Very large and/or complicated models may also be unwieldy and opaque when coded directly, and an important resource are general-purpose ODE solvers which can translate and solve models specified using a markup language, allowing modellers and readers to concentrate on the model rather than its implementation. For example, the odeSD tool [48] provides a model conversion framework for SBML models and integrators implemented in the Matlab and C languages.

Like the bioinformatic analyses described above, simulations of biological systems and parameter inference algorithms often involve a stochastic element [49–51] and this clearly presents a challenge for exact replicability. Some differences between the original and the reproduced results are expected and it is helpful to have an idea of what the key results are, how to measure agreement between the original and reproduced work, and how close that agreement is expected to be. This can be arranged by repeating the simulation or sampling process multiple times and reporting the variation in the outcomes: see, for example, Figure 8 of [52]. If the outcome of interest is a qualitative pattern of behaviour, it may be appropriate to record the proportion of runs on which a particular pattern emerged – but of course, this depends on a clear computational statement of what defines the pattern.

#### Reproducibility in data analysis, simulation and inference

We considered under the heading of replicability the quantification of software-related uncertainty. The other aspect of uncertainty when parameters are to be inferred is that which is inherent in the data. The theory of inference and error analysis already provides an established framework for understanding this distinction between probability as representing empirical variation versus uncertain knowledge [53]. Estimates should always be reported with an associated uncertainty. If an investigator reproduces the result using a new dataset, agreement between the two estimates can be assessed using their respective uncertainties.

Parameter inference studies often lend themselves to attempts to reproduce the same conclusion by a different method. For example, inference by maximum likelihood could be repeated in a Bayesian framework to investigate the effect of incorporating prior information. By trying to reach the same conclusions in a different way the investigator gains more confidence in the result – and also a deeper understanding of the system and its behaviour.

#### Extensibility in data analysis, simulation and inference

A clear statement of the key results to be reproduced is recommended for extensibility as well as reproducibility. Any extended model should agree with the original in the limit where the two are equivalent. Stating what is meant by equivalence is a good discipline for understanding the properties of the model, and also a way of making a model more accessible to others for extension.

Because other researchers may want to reproduce some elements of a piece of work while changing others, it can be helpful as in the bioinformatics example above to write code in a modular way – although the most useful way to achieve modularity is another area of discussion [54]. This is good practice in any case and of course, it does not only make extension easier for others – it will be easier for the original author as well. The history and evolution of a project is also important information for facilitating reuse of code. Version control systems like git [55, 56] provide a ready-made framework for handling this information and making it available for the developer, the user and others who might contribute to the project, especially when combined with online facilities such as GitHub. Once again, following standard good practice also improves reproducibility and extensibility.

#### Case Study 2: Linking data analysis, mechanistic models and inference using Jupyter notebooks

Our first example of a tool facilitating reproducibility in data analysis, simulation and inference studies is the Jupyter notebook (formerly IPython notebooks [57]). The notebook is “a web application that allows you to create and share documents that contain live code, equations, visualizations and explanatory text” [58]. Notebooks have emerged as an increasingly popular tool for aiding reproducible research, and are just one example of a family of integrated analysis and presentation tools which also includes Mathematica notebooks [59] and the R tools knitr [60] and Sweave [61]. The Jupyter project supports over 40 programming languages, but we concentrate on its use as an interface for IPython [62], which has been developed with scientific analysis in mind.

##### Jupyter notebooks: replicability

Jupyter notebooks provide, in essence, a convenient, shareable, executable lab book. A number of examples in various languages can be found online [63]. As a general caricature of their use, raw data can be received in a simple format and must initially be cleaned, processed and possibly subject to some exploratory analysis. A first step to enabling simple replicability of this processing is to record each step. In Jupyter notebooks, commands are recorded completely and may be grouped into convenient, somewhat autonomous blocks so that key steps can be checked, tested and re-run separately or replicated independently. Any important or illuminating checks may be left in the notebook and exactly duplicated by others by executing the appropriate cell. Rich-text documentation is also available using markdown, HTML and/or LaTeX formatting commands, which greatly aids communication.

##### Jupyter notebooks: reproducibility

Computational researchers generally aim to go beyond empirical data analysis to develop mechanistic mathematical models. Checking and critically reviewing these computational equivalents of an experimental protocol is an important aspect of reproducibility because it allows others to propose changes and alternatives and inspect the differences in results. The Python language is a good candidate for implementing mechanistic models, having a large and growing number of packages for scientific computing and thus offering a common environment for data processing and model implementation. However, it also has some drawbacks. For example, the notebook format does not prevent poor coding practice: it is perfectly possible to prepare a poorly commented, badly organised Jupyter notebook. Some tools and packages may perform badly because of Python’s interpreted nature. This may then require the use of ‘lower level’ programming languages, either separately or called within Python, so that some of the valuable transparency of the setup is lost.

##### Jupyter notebooks: extensibility

Jupyter notebooks offer a dynamically updating, error-correcting tool for the publication of scientific work, facilitating extensibility in a similar way to online manuscript archives such as the arXiv [64] and pushes for “post-publication peer review” and article comment sections [65]. However, single notebooks can become unwieldy for large analyses. To allow reuse and extension of analysis components, notebooks can be separated into book-like or hyperlinked formats, allowing a higher-level division into chapters while still maintaining lower-level manipulability and testability at the cell or single line of code level. Importantly, the Jupyter project supports several languages, and this cross-language support is being actively developed.

It is important to note that stacking incremental improvements on existing code may be useful only in the short term. In the long term, an inflexible adherence to this way of working may prevent progress and limit software performance, and a completely different approach may be called for. In order to aid true extensibility, it is important to clearly explain the algorithms implemented in the software. Ultimately, it is the concept that is essential for future implementations, even more than the code, and both must be made easily available.

We find that, as typically used, Jupyter notebooks are a useful step towards replicable and reproducible research. They have even been used to help create “executable” papers [66, 67]. On the other hand, the integration of the development, exploratory and presentation phases of analysis can be difficult for reasonable-sized problems. Ironically, the limitations of analysis scripts return – a single, executable form capturing all phases of the analysis may be provided, but this may not be as comprehensible as a presentation of one of the phases by itself. Furthermore, reusability and further development by other users can be compromised by the goals of unified presentation of results. Finding the proper balance between these competing demands will be of increasing importance. A set of minimum information standards or documented best practices for formatting and sharing scientific notebooks, in addition to the continuously improving functionality, would be a useful step in this direction.

#### Case study 3: the Chaste ‘paper tutorial’ approach

Our final example relates to the Chaste (**C**ancer, **H**eart **a**nd **S**oft **T**issue **E**nvironment) package for simulations of biological systems. It is designed as a set of C++ libraries to facilitate building models and running simulations for a range of application areas [68, 69].

##### Chaste: replicability

Due to Chaste’s intended use as a library of functionality upon which users can build, considerable effort has been devoted to checking that the software will run with consistent behaviour on a range of systems. It is open-source software, and has been installed by many users on a range of systems, from personal laptops to high performance clusters, following a range of installation guides. This is still often a difficult process, and so we are investigating the use of Docker to make it easier for new users to get started. This is unlikely to be a feasible route for use on a high-performance computing resource, however, where third-party dependencies specific to the hardware are needed for best performance.

Chaste also comes with an extensive test suite to verify that the behaviour of individual components and whole simulations is unchanged across different systems, and across versions of the software itself and its third-party dependencies. These tests are regularly run automatically on a range of setups. It might therefore seem that replicable behaviour is highly likely. However because we are working with floating point arithmetic, results are only ever tested up to a developer-defined tolerance, so even this is not exact replication. Rather, the tolerances provide a specification of how much variation is expected in particular results. The reliability of these tests also depends on the care with which the developer has chosen tolerances – often these are set by ‘feel’ since a formal expected error cannot be derived. As in previous examples then, we have a spectrum from replication to reproduction.

##### Chaste: reproducibility

Automated tests such as those described above are most commonly adopted to check the behaviour of low-level code components. In Chaste, however, the same framework is also exploited for running the simulation experiments leading to published results. It is thus (in principle) simple for researchers to augment their simulations by comparing the results to those achieved during the initial run. This can provide useful documentation for subsequent users in terms of what the key results are, and what is viewed as being sufficiently similar. A “literate programming” [70] system that we have termed “paper tutorials” is also provided, where comments can be added to the source code which trigger the creation of a rendered version on the Chaste website. Two papers in particular [71, 72] make use of both these features, and there are several more [73] which exhibit varying degrees of documentation and comparison to reference results. As ever, while having the technical framework to do this is better than starting from scratch, a technical solution does not guarantee that it will be used to full effect to improve the science done. The hardest part is for the researchers to define what the key features of their results are, and then implement checks for these in the software.

##### Chaste: extensibility

Chaste combines many of the features noted in the other case studies. Provision of the rendered simulation experiments with full source code and installation instructions greatly facilitates users in adapting what has been done to new scenarios. Indeed, this is often the approach that the developers take themselves! Extensive tutorial material, briefer “how-tos” and in-line documentation help to guide new user/developers in determining where and how to make changes in order to achieve the desired results. Despite this, some changes remain easier than others, and given the size and complexity of the software it is challenging to extend beyond problems similar to those already addressed, especially for a new user. There are costs to producing well documented and usable software, and the balance between focussing on the next publication using new methods, and implementing those methods in a way accessible to others, is often not easy to judge.

## Discussion

In our attempt to go beyond general discussions of replicability and reproducibility by considering case studies from our own research experience, we immediately faced the problem – across different contexts – of defining the specific features of a result that we aim to reproduce. Exact numerical equivalence is rarely of interest. Instead, there is in general some anticipated tolerance on quantities of interest. We saw that frameworks for quantifying and describing uncertainty already exist in statistical interpretations of probability, but these are underused and often only employed to present uncertainty related to the limitations of data, when they could also be applied to uncertainty associated with stochastic elements of analysis, simulation or inference methods.

We noted a general trend towards dynamic, updatable environments including Jupyter, knitr and Sweave, which move beyond static publications [58, 60, 61]. These new formats provide executable, interactive lab books and are more reflective of the process and increasingly collaborative nature of modern science. The trend can be facilitated by platform-independent tools such as virtual machines and web-based applications, although these tools can equally foster a ‘black-box’ approach which does not encourage, or even allow, the user to probe the details of how the software works. It is becoming clear that traditional research papers and peer review processes are an incomplete medium for communicating the context needed to fully replicate or reproduce a scientific result, and post-publication peer review is allowing the community to question and comment on published work. However, a tension arises between complete description and clear communication. When so much detail and information is available, how can authors guide the reader towards the key message of the study? And how should we integrate best practices for code development and the presentation of results? Complete documentation only permits reproducibility: clear communication is also required to motivate and simplify it, and the challenge lies in balancing the two. In a similar vein, extensibility presents a problem of balancing demands of clarity against flexibility and reuse. The analysis script for a figure in a publication, for example, should focus more on clarity of presentation than modularity for future extension. Core numerical/simulation libraries, on the other hand, need to focus primarily on ease of reuse, incorporation into larger systems, easy replacement of modules, and the like.

Our observations also raise the question of how good reproducibility practice can be made feasible and attractive. In a world where researchers are judged mainly by numbers of publications, citations and funding income, how should we facilitate and incentivise good practice in a cost-effective way that is achievable for busy researchers? One idea might be a reproducibility “seal of approval” which individual researchers, groups or departments could be awarded and which would improve chances of attracting funding, in a similar way to the Athena SWAN awards [74, 75] for commitment to equality and diversity which are becoming required by some UK funders. Minimum Information reporting guidelines would provide a helpful starting point for defining the criteria for such an endorsement. Scientific journals could stipulate adherence to minimum information checklists as a requirement for publication. An official standard endorsed by the community and by funding bodies might help to reassure researchers who are worried about sharing code and data. Support structures such as Software Carpentry [76, 77] also exist in part to address these concerns and introduce best practices to as many researchers as possible, and a commitment to this kind of education might form another part of the standard.

Encouragingly, the trends we have identified – increasing standardisation of data formats and reporting guidelines; clearer definition of the key features of a result to be reproduced, and more use of dynamic publishing tools – suggest that good practice is not only becoming increasingly common and valued in the computational biology community but that it is in fact becoming intrinsically linked with how research is published and communicated. Aspects of standard good practice in software development [7, 8] (version control, modular design) also make research more reproducible. However, although appropriate tools for replicable research are increasingly available, researchers seldom provide advice to their readers about how to quantify confidence in their results. Improving on this state of affairs will require convincing researchers of the benefits of allowing true reproducibility – in addition to replicability – of their results.

## Conclusions

**Fig. 1:**
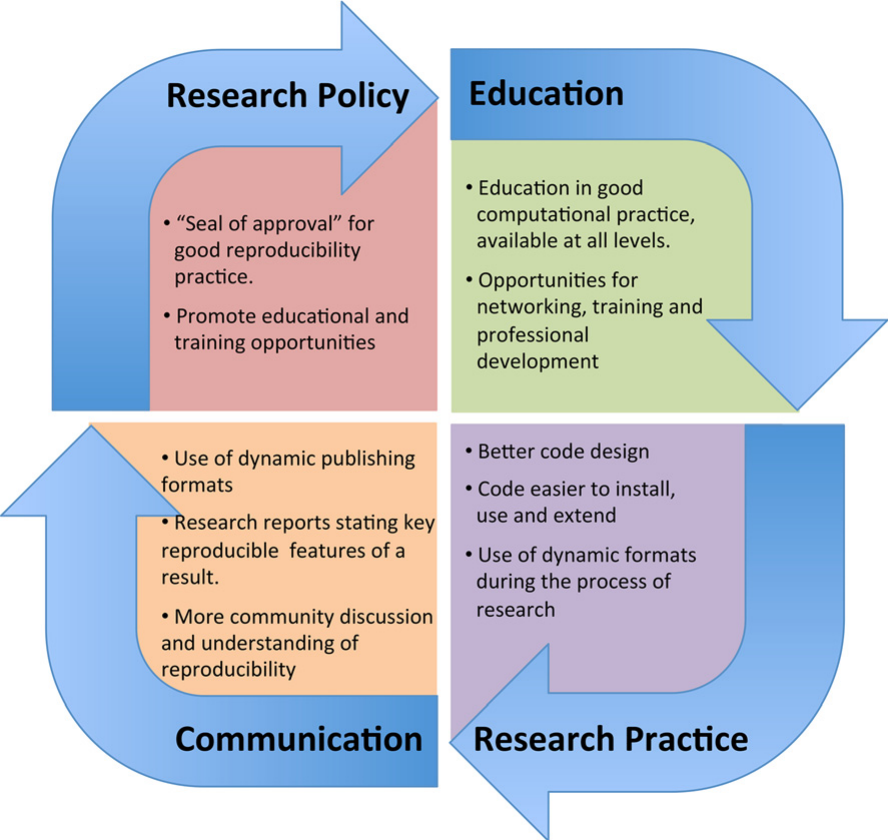
A “virtuous cycle” of good reproducibility practice

We conclude by offering two summaries of our findings. First, Table 1 brings together the checklists, minimum information guidelines, exchange protocols and software tools described in the manuscript. As well as providing a useful reference for researchers seeking appropriate tools and standards, we hope that it will highlight gaps which could be filled by checklists to be developed by experts in the relevant specialities. Second, Table 2 presents a set of recommendations for improving software practice in computational biology, gathered from our experiences described above. The recommendations are for implementation by developers, researchers and the community as a whole – and we emphasise that most researchers fall into all three of these categories. Figure 1 illustrates how these recommendations could form a virtuous circle, whereby an effort on behalf of the whole community and improved education promote good practice and good communication, leading back in turn to an understanding of the importance of reproducibility and a stronger agenda within computational biology.

**Table 1:** A summary of tools and standards for reproducible computational biology

Area/activity	Tools and standards
Umbrella projects for guidelines and standards	MIBBI [11], COMBINE [45]
Training	GOBLET [34, 35], Software Carpentry [8, 76, 77], Data Carpentry [78]
Data exchange and analysis	PSI-MI [12, 13], BioPAX [14, 15]
Model exchange and annotation	MIRIAM [43], COMBINE standards [45] e.g. SBML [46]
Simulation experiments	MIASE [44], SED-ML [47]
Notebooks	Jupyter [57, 58], Mathematica [59], knitr [60], Sweave [61].

**Table 2:** Recommendations for improving reproducibility practice

Software developers can:
• Use well thought-out and appropriate principles of modularity in designing software. • Provide practical, comprehensive advice on installation. Check it by installing software on commonly-used systems, or simplify it using a platform such as Docker. • Provide code annotation and manuals in multiple, accessible forms with different levels of detail.
Computational researchers can:
• Make use of dynamic and/or updatable formats for publishing research, where appropriate. • Ensure they provide all details of how an analysis was carried out, including providing all the code and data necessary to reproduce a result. Context-specific minimum information guidelines can provide useful checklists. • State explicitly what are the key features of a piece of published work, how to measure agreement when the work is reproduced, and how close the agreement is expected to be.
The computational biology community can:
• Introduce a “seal of approval” for good reproducibility practice including adherence to reporting checklists, which could be awarded to labs, individual researchers or particular pieces of software or research. • Require adherence to appropriate minimum information checklists for publication in peer-reviewed journals and through other channels. • Promote and campaign for education in good computational practice for scientists of all backgrounds, from undergraduate to professorial level. • Provide structures and opportunities for networking, support and professional development of computational researchers.

## Methods

This work was carried out during the Software Sustainability Institute (SSI)/2020 Science Paper Hackathon, 10–12 September 2014. The authors had identified case studies from their own research experience, to which they felt the topic of reproducibility was relevant. During intensive discussions over the course of three days they compared and discussed the case studies with the aim of drawing out common themes from across computational biology and developing a framework for understanding reproducibility in the computational/systems biology context. An early version of the paper was drafted during this stage. Following review and comments from another participant in the hackathon (working on a different project), the ideas and the manuscript were refined and clarified over the following months.

## Abbreviations

BioPAX, Biological Pathway Exchange; Chaste, Cancer, Heart and soft tissue environment; COMBINE, Computational Modelling in Biology Network; eFORGE, Functional Element Overlap analysis of the Results of Epigenome-wide association study Experiments; EWAS, epigenome-wide association studies; GOBLET, Global Organisation for Bioinformatics Learning, Education & Training; HTML, HyperText Markup Language; MIASE, Minimum Information About a Simulation Experiment; MIBBI, Minimum Information for Biological and Biomedical Investigations; MIRIAM, Minimum Information Required in Annotation of Models; ODE, ordinary differential equation; odeSD, second-derivative ordinary differential equation integrator; PSI-MI, Proteomics Standards Initiative Molecular Interactions; SBML, Systems Biology Markup Language; SED-ML, Simulation Experiment Description Markup Language; SWAN, Scientific Women’s Academic Network; UK, United Kingdom
